# Bibliographical Notices

**Published:** 1854-01

**Authors:** 


### BIBLIOGRAPHICAL NOTICES.

PRIZE ESSAY. *The Surgical Treatment of certain Fibrous Tumours of the Uterus, heretofore considered beyond the resources of Art.* By WASHINGTON L. ATLEE, M. D., of Philadelphia. *Extracted from the Transactions of the American Medical Association for the year* 1853, *for private distribution.*

"Palmam qui meruit ferat!"

In the above essay, for which we are indebted to the author, Dr. Atlee details the surgical treatment of 14 cases of certain fibrous tumours of the uterus, which have heretofore been considered inaccessible to the knife or not amenable to curative measures.

As the subject is a highly important one and the treatment claimed to be novel, we shall endeavor to give an accurate, though brief synopsis of all the cases mentioned.

The tumours are classified as follows:

1. EXTRA-UTERINE, or surface tumours.
2. INTRA-UTERINE, or cavity tumours.
3. INTRA-MURAL tumours of the uterus.

Of these several forms, he gives the positive and differential diagnosis and a concise but lucid account of the symptoms of each in their several stages.

Under the head of "Treatment" especial attention is called to a fact which is considered of great importance. It is "that the excessive hemorrhages, which sometimes occur, arise, not from the uterus itself, but from the vessels of the membrane which cover the tumours." To arrest these, we are advised to pass the bistoury along the vagina into the cavity of the uterus, and make a very free incision into the most exposed portion of the tumour."

Regarding the prognosis and duration of these affections, when not malignant, we regret that we do not find any particular statement. We consider this to be a most important omission, as on such statistics alone must be determined the necessity of surgical interference. The only observations on these points are the following, which as illustrative of the views of Dr. Atlee on the pathology of these tumours, we feel sure, must have exerted great influence over his mind in his decisions.

"Besides the above classification, there is another distinction which it is important to bear in mind in connection with the prognosis and the period of treatment. I believe there is, at times, a tendency in fibrous tumours of the uterus to take on cancerous action. I know a diversity of opinion exists on this point among pathologists of eminence, but the results in certain cases under my own care lead me to think that the true fibrous tumour will occasionally degenerate into medullary tissue. It may be that *scirrho-cancerous* tumours have in these instances been mistaken for *fibrous* tumours, as the latter, by excellent observers, are deemed incapable of becoming cancerous. Yet they were diagnosed to be fibrous, and they possessed all the physical characteristics of such tumours. If they were cancerous tumours, then I know of no method of distinguishing them in their early stage from fibrous tumours, except by the microscope, and the microscope in many of these cases, cannot be employed as a diagnostic means. In some cases the exploring-needle will enable us to get sufficient of the structure to be submitted to the test of the microscope. If, however, I am correct in the opinion that the true fibrous tumour occasionally degenerates into cancerous disease, then it is important to know the symptoms which designate this change. So long as it remains hard and indolent, it does not greatly interfere with the general health; but so soon as it begins to soften or degenerate, it rapidly impairs the health and terminates in death. Hence the necessity of distinguishing this point, and the urgency of early treatment."

The fourteen cases are then detailed. Of these 7 are Intra-uterine, 6 Intra-mural and 1 Extra-uterine or pelvic.

Of the 1st or intra-uterine class three deaths are reported, one from inflammation of the lungs, one from erysipelas and one suddenly from disease of the heart. In the 1st (Case I.) a tumour of the supposed weight of ten pounds was removed; the final operation for the excision of a mass of the size of an orange, taking place on the 9th of July. Two days previous to this, after an alarm referred to, the patient had been seized with cough, followed by copious expectoration. The pneumonic symptoms gradually increased and the patient died on the 16th; seven days after the last operation, and nine after the first occurrence of the thoracic disease.

Before the operation, menstruation had been regular, with only slight menorrhagia, and health tolerably good. The autopsy showed, that all the viscera of the thorax, abdomen and pelvis were perfectly healthy, excepting the lungs.

In the case which died from erysipelas, (Case IV.) gastrotomy was performed, the tumour being supposed to be extra-uterine. The patient recovering from this, an attempt was then made to remove it per vias naturales, when erysipelas supervened, ending in death. The patient's health, before the operation, had been slightly disordered.

The third death (Case VII.) occurred suddenly from disease of the heart. Syncope came on whilst she was being operated on. From the prostrating effects of this she partially recovered, but died two days afterwards. During life, the patient had been anemic, from too profuse menstruation. The autopsy revealed a healthy state of all the viscera, excepting the heart, which had undergone the fatty degeneration.

Of the Intra-mural class, six in number, two deaths are reported—one from peritonitis, (Case X.) in which, after apparent convalescence from the effects of an operation, peritonitis ensued, ending fatally. The autopsy revealed malignant disease; the whole uterus being converted into medullary matter, with no perceptible muscular fibre.

The patient's previous health, excepting at the menstrual period, had been pretty good.

And one from anemia. (Case VI.) In this instance, the patient was bloodless from repeated floodings, and her life was considered to be in imminent hazard from present hemorrhage. The bleeding ceased immediately on operating. After this, the author says, Page 61: "4, P. M. Saw the patient in company with Dr. Grant. It being feared that the chill, fever and oppressed breathing arose from the irritation of the putrefying mass in the pelvis and abdomen, it was decided to remove as much of the tumour as possible." This being done, it was found to fill a large wash-basin, and in its degenerated state weighed five pounds He again says: "10, P. M. The removal of the tumour did not appear to afford relief to the symptoms. Respiration became more and more labored—the pulse was small, though not frequent; there was considerable restlessness and jactitation, and the patient was evidently sinking. Anodynes and stimulants did not relieve her, and she died at 2 o'clock."

"The opinion of the medical gentlemen present, as expressed by a certificate, was that she died from *anæmia*."

Of recoveries, nine cases are detailed, four Intra-uterine, four Intra-mural and one Extra-uterine or pelvic.

Of the four Intra-uterine, one (Case III.) recovered after repeated operations, in which a tumour of the supposed weight of six or seven pounds was removed. Two years and a half afterwards, there was a reproduction of the tumour, which was again removed in December, 1851. From this last operation, the patient did not rally for two or three days, the prostration being great, though not from hemorrhage. Some of the symptoms were attributed to the long continued use of chloroform.

We should mention, however, that some years previous to the first operation, this patient after repeated and excessive floodings, had been relieved, by the efforts of nature of a polypus which filled the whole pelvis.

Previous to operation the patient had suffered much from excessive floodings.

*2d recovery*, (Case V.) In this instance the uterus was distended to the size of full pregnancy; by repeated operations, the whole tumour, weight eight or nine pounds, finally came away. The degeneration of the mass, caused by its section and the admission of air into its substance, produced an immense and very offensive flow from the vagina. This was accompanied with irritative fever and considerable oppression over the chest, with some difficulty of respiration. These symptoms being relieved, the patient finally regained her health perfectly, and engages in all kinds of household labor. The patient's health, previous to operation, had been much shattered.

*3d recovery*, (Case VIII.) A prominent tumour, weight seven or eight pounds, was here present. After repeated operations, in which very little of the tumour was removed in substance, an offensive discharge, the result of its destruction through admission of air, came on, by which the whole mass was finally got rid of. Her health, three years afterwards, was perfectly restored. Before the operation, excepting symptoms of erysipelas, by which she once had been attacked, she had always enjoyed good health. Occasionally there had been slight leucorrhœa. All her functions were in good order.

*4th recovery*, (Case XIII.) An intra-uterine tumour attached to the cervix. The operation in this instance seemed to have had but a temporary effect, as in less than two months afterwards Dr. Atlee was informed by letter, dated December 18th, 1852, that there was but little change "until the last few days, and that has been quite a flow of blood, say from eight to ten ounces, within the last twenty-four hours." Dr. Atlee remarks: "The greatly enlarged abdomen of this patient by adipose tissue, rendered it impossible to know anything about the development of the tumours upwards. I am inclined to think, however, that they projected from the interior of the cervix, and that they originally were schirro-cancerous. After their removal the same disease developed itself at the point of attachment, and in the os tincæ. The final result of the case is not yet determined."

INTRA-MURAL TUMOURS.—Of these four cases are stated to have recovered by operation. The first (Case II.) was an instance of a tumour of the weight of four or five pounds, developed in the posterior wall of the uterus, and expanding that wall into a cyst inclosing it. This, by operation and the decomposition of the mass which afterwards ensued, gradually wasted away, leaving no vestige of the disease. Four years afterwards her health is stated to be most perfect. It had been much shattered previous to operation, by repeated attacks of flooding and severe pain.

*2d recovery*, (Case IX.) Here the cervix uteri was bent against the tumour at an acute angle. After repeated operations, the tumors diminished. The corpulency of the patient and the high position of the mass, however, finally determined Dr. A. to discontinue all operative proceedings. The remains of the tumour are still discoverable by examination per vaginam. Her general health is stated to be pretty good, and much improved since the operation.

*3d recovery*, (Case XI.) The whole anterior wall of the uterus was nodulated, in this instance, from the fundus to the os tincæ, and the patient was perfectly anemic from excessive floodings. One year afterwards, September 1852, the patient, it is said, had much improved in health and had had no return of the hemorrhage. In his remarks on this case, the author states that "The general health and constitution also had become greatly impaired, the cancerous diathesis seemed to have been developed, and even the tumours had softened so as to stain the finger with a greyish encephaloid matter; yet the operation was followed by restoration to health.

*4th recovery*, (Case XIV.) Intra-mural tumour of about seven or eight pounds, developed in the anterior wall of the uterus and expanding that wall into a cyst inclosing it, was here removed. By repeated operations during the months of March and April, 1853, the mass was entirely destroyed. On the 1st of May, the last day she was seen, it is stated that she had rapidly improved in health and was making preparations to return home. Previous to being operated on, the patient flooded largely at each menstrual term.

Extra-uterine or pelvic tumour. Of this but one instance (Case XII.) is recorded. The patient had suffered from bearing down and pressure, and frequent calls to urinate. Bowels regular and never had had leucorrhœa. The diagnosis being obscure, it was agreed, if the patient should insist upon an operation, to open the abdomen and be governed accordingly. Gastrotomy being performed, the tumour was found in the posterior and inferior part of the cavity of the abdomen, and not removable. From this operation the patient recovered. Three months afterwards, March, 1852, Dr. Atlee says, "The patient was much more comfortable after the operation of gastrotomy, but regretted that she still had to endure the presence of the tumour."

Having ascertained, by opening the abdomen, its exact location, it was operated on through the vagina. Five months afterwards, the patient's abdomen was still large, but in all other respects she enjoyed good health.

To save our readers the trouble of summing up these cases, we will state that, of the five deaths, three occurred in persons who were in the enjoyment of pretty good health previously; one patient was anemic from too profuse menstruation, and one bloodless from excessive floodings. Of the recoveries, six were complete, and three incomplete. Five of the former were in bad health previous to operation. In the other instance the patient's health was good. Of the latter or incomplete recoveries, one was not improved in health by the treatment; the disease being again developed and thought to be cancerous. In the two others, though the health is stated to be improved, there still exist remains of the tumour.

From mistakes in diagnosis, gastrotomy was performed in two instances, and in the case which died from peritonitis, the autopsy revealed the existence of most extensive malignant disease. We presume the patient would have been let alone if this condition could have been previously ascertained.

Dr. Atlee's operative proceedings in the above cases consisted, except where the tumour was pelvic, in the active administration of ergot to produce uterine contractions and to dilate the os tincæ. When the latter would not yield, he would nick it in several places with a bistoury; often extending these through the cervix and cutting with the same instrument deeply into the uterine tumour. These operations he would frequently repeat, and labor being artificially induced by the ergot, with the bistoury, crotchet, forceps, hook and his fingers, he would sever connections, cut, break up and tear away the mass in substance. The destruction of vitality of what remained and its disintegration by the admission of air produced a profuse flow of dead and mostly fluid matter, by which means the residue would be discharged.

In one instance, the whole length of the uterus was incised. The account is as follows:

"Every means having been employed by Dr. Harry, without effect, to arrest the hemorrhage, I proposed to lay open the hypertrophied portion of the uterus, with the double object of arresting hemorrhage and destroying the disease. The proposition having been accepted, I passed the long bistoury three inches into the uterus, and turning its edge against the anterior hypertrophied wall, made an incision along its whole length, sinking the knife deeply into the substance of the uterus. Immediately the nodules diminished, and the mass became softer. The hemorrhage at once ceased, and no bleeding followed. On withdrawing my finger, there adhered to it a grayish kind of matter. I could still feel another nodule to the left of the incision, but not larger than the smallest filbert, which I also incised."

Before we proceed to make any remarks upon Dr. Atlee's paper we must confess our surprise in not finding any mention in it of Dr. Gilbert's case of "fibrous tumour" of the uterus, published in his "Contributions to Surgery," in the Jan. No. of the American Journal, 1851. The functions of defecation and urination being much interfered with in this case by the position of the tumour, Dr. Gilbert, after much reflection, resolved upon the following operation. Bringing down the uterus with a tenaculum, "he made an incision, but found the cellular tissue so condensed that it was manifest the tumour could not be removed in this way." He proceeds: "I then made very free and large incisions into the tumour with the expectation that the imperfectly organised mass would become atrophied by the discharges which must ensue from these incisions, since union by adhesion or granulation could not here take place. Success beyond my most sanguine expectations followed this procedure; the tumour diminished in size, and I have not been called to prescribe in the case or to pass the catheter during the last five years."

This operation occurred in the spring of 1845, and we are informed was the first of the kind ever performed for fibrous tumours of the uterus.

Our readers will form their own conclusions, regarding the nature and results of Dr. Atlee's proceedings. To some, they may prove a guide; to more, we trust, a warning. Our own opinion is that such operations are not in accordance with principles of sound surgery. Conscientiously, we consider the remedy to be more dangerous than the disease. Women live with such tumours for years; and if the disease be malignant, we think for many reasons they had better be left to die a natural death. Nor can we imagine, from the high estimate in which we hold Dr. Atlee's surgical abilities and foresight, that the average results could be more fortunate in other hands.

Should hæmorrhage induce us to operate? Science has many resources in such cases and when these fail, nature, more kind, sometimes steps in and accomplishes a cure for us. (See Case III.) We have been assured by Prof. Hodge, that during the last 25 years, but one death has occurred in his practice by hæmorrhage from an intra-uterine tumour.

Add to these considerations, the fearful evils which may result from mistakes in diagnosis; which must sometimes occur, even with the most experienced; and we are strong in the conviction that the profession will agree with us, in stigmatising such operations as the very opprobria of Surgery.

*On the Etiology, Pathology, and Treatment of Fibro-Bronchitis and Rheumatic Pneumonia.* By THOS. H. BUCKLER, M. D., formerly Physician to the Baltimore Almshouse Infirmary. Philadelphia. Blanchard & Lea.

The perusal of this treatise has given us much pleasure. It is the production of a cultivated and ingenious mind. Its leading object is "to point out, as clearly as possible, the distinctive characters of fibrous or rheumatic inflammation of the bronchial tubes, and at the same time to show the differential diagnosis between it and ordinary catarrh; the word rheumatic has therefore been affixed to the term bronchitis, for the purpose of showing at the outset that it is intended to treat of a distinct affection, which, for want of proper anatomical accuracy as to its true seat, has been most singularly confounded with inflammation of the mucous membrane of the bronchi.

"The next object is to show that there exists a form of pneumonia which is never idiopathic, but occurs as a secondary lesion, and is always symptomatic of, and directly dependent on, pre-existing fibrous bronchitis. It is farther intended to point out the relations which the foregoing pathological conditions bear to general rheumatism and to rheumatic endocarditis, and to show that ordinary pneumonia, simple mucous catarrh, and fibrous bronchitis, with rheumatic pneumonia, often happen in the same lung as distinct, but still contemporaneous and concurrent, affections, and that where this is the case, therapeutic attention to the rheumatic element is often of vital importance to the safety of the patient."

Dr. Buckler thinks "it very remarkable that the differential diagnosis between mucous and fibrous inflammation of the bronchi should have remained without elucidation until this time, particularly when it is remembered that the relation which these two tissues bear to each other is so like that of the two similar tunics of the eye, the sclerotica and conjunctiva. Probably the reason of this neglect is, that fibrous bronchitis is so often complicated with mucous catarrh and pneumonia."

"The symptoms most strikingly characteristic of the acute variety of rheumatic bronchitis, are profuse, irregular sweats, inordinate sensibility to cold, transient flushings of the face, and either a transient or paroxysmal and unproductive cough." "The disease has to be made out in most cases, solely by the method of exclusion, as far as the auscultatory sounds are concerned, as these are entirely negative, with the exception of occasionol sibilant râles."

An excellent chapter on the vascular mechanism of the pulmonary circulation is given us, in which the separate functions of the two circulations, the pulmonary, and the bronchial or nutritious, are strongly insisted upon. Following this, we have some remarks upon rheumatism and the rheumatic element, and the whole is concluded by cases illustrative of his peculiar views and treatment.

Page 125, he says: "In the acute variety, uric acid and urate of soda are found in excess in the urine, unless the kidneys refuse to secrete them, and then the absence of these salts in the urine is generally an index of their superabundance in the blood."

It is probable that in such cases, the absence of urates is due to their retention in the lungs, not that the kidneys refuse to secrete them, and that when reabsorbed into the blood, they again appear in the urine. Mr. Beale, in his paper "On the Diminution of Chlorides in the urine in Pneumonia" has satisfactorily shown, by a chemical examination of the sputa, that there is a determination of this salt to the inflamed lung which ceases when resolution occurs. We think the subject is worthy of investigation, as it will throw much light on conditions hitherto unexplained.

Dr. Buckler has conferred a benefit on the profession by the publication of his views, and we heartily commend them to the perusal of our readers.

---

*A Text Book of Anatomy and Guide in Dissections.* By WASHINGTON R. HANDY, M. D., Professor of Anatomy and Physiology in the Baltimore College of Dental Surgery. Lindsay & Blakiston, Philadelphia.

Dentistry is assuming a more elevated position than it has heretofore occupied. No longer regarded as a mere manipulatory art, requiring dexterity and some experience only for its successful practice, its professors are now earnestly endeavoring to elevate it to the dignity of a science. As evidences of this may be noted the publication of several Journals and the establishment of four Colleges in different places throughout our country. As another token that "the schoolmaster is abroad," may be mentioned the issue of the above "Anatomy," which though it may be considered supererogatory in view of the many admirable treatises already extant, yet as a proof of an independent and self relying spirit deserves and should receive our hearty commendations.

In the preface, the Author says that "Dental students are slow to see and feel the necessity of a knowledge of any more of Anatomy, than so far as the teeth and their immediate connections in the mouth are concerned, and to go beyond this is thought rather a waste of time, and entirely foreign to the practice of the profession they design to pursue." To correct this false and dangerous sentiment was the chief reason which induced Dr. Handy to write his "Text Book."

The plan of the work after giving a general outline of organization, divides the subject into four parts. The 1st describes the Elementary tissues of the body. The 2d that of the Head and its organs, in their functional order and dependency, and is then made complete by showing the anatomical and physiological *Relations* of the *Mouth* with the different parts of the *Head.* The 3d, the Trunk in the same order and dependency, "completed in the same manner, by demonstrating the *Relations* of the *Mouth* with its several organs, viscera and functions. The 4th part comprises the Extremities which are demonstrated in the usual way. About seventy pages are devoted to the description of the Teeth, their histology, and comparative anatomy. This part of the work we commend to the attention of dental students and practitioners, as containing a valuable and reliable resumé of the present state of knowledge on this subject.

The unusual prominence given to the relations of the mouth with different parts of the body, is a most singular feature in this work. For instance, under the head of "Anatomical and Physiological relations of the Mouth with the Extremities," we find the following. "Under this head we shall only refer to the well known fact of trismus or locked jaw resulting from injury to the toes, thus establishing a relation between the mouth and inferior extremities. This relation, in all probability, occurs through the spinal marrow and fifth pair of nerves.

The relations of the mouth with the superior extremities, are no doubt, equally close and important with those of the inferior extremities."

Unquestionably they are, and the same remark will apply to its relation with every part of the body; scalp, fingers, nose, and wherever we have a surface supplied with nerves; but why a particular division of the book should be allotted to such self-evident statements is incomprehensible.

The book is admirably printed in good clear type and ample margin. We regret that we cannot commend the wood cuts, which were executed in Baltimore.

---

*Report of the Standing Committee on Surgery, read before the Kentucky State Medical Society, Oct.* 1853. By JOSHUA B. FLINT, Professor of Surgery in the Kentucky School of Medicine.

Dr. Flint's report is devoted to the consideration of the undue importance attached to operative surgery, "together with some of the incidental evils connected with that mal-appreciation of the therapeutic resources of surgical practice."

"The passion for operating, always sufficiently conspicuous in miscellaneous practice, has displayed itself, at different times, with appalling recklessness, in the conduct of specialities, and gathered its bloody laurels under favor of a present infatuation or panic respecting a particular form of disease. The *circulatores* of the middle ages, and the *emasculatores* of the eighteenth century, present remarkable illustrations of surgery run mad, in times past, while the *myotomists*, the *ovariotomists*, and the *womb-burners* of our own day, may fairly contend for the distinction of furnishing a modern parallel.

A tempting field for indulging the 'rage for cruel and bloody operations,' which Dr. Lee says 'has spread far and wide in England, and threatens to pervert and corrupt the sound and fundamental doctrines of British practice,' is found in the treatment of morbid growths and malignant degenerations presenting themselves in the form of tumors, on the surface or in the cavities of the body. In the management of these diseases, a class of questions arises, the determination of which often involves the character of surgery in general, as well as that of the practitioner immediately interested, and exhibits, in signal contrast, the sound, conservative surgery of science, and that flippant, reckless counterfeit of it, that rests in artisanship.

In the midst of these perplexing questions of diagnosis and treatment, with which such cases often confound the most competent practitioner, the true surgeon pauses, watches, consults, palliates, and perhaps cures —the hero of the scalpel cuts the gordian knot, and with it, perhaps, the life-thread of the patient."

"No grave operation should be undertaken upon a mere vague *hope* of success. The prudent and conscientious surgeon will act upon nothing short of a reasonable probability of definite and sufficient advantage to the patient, as a compensation for the pain and injury inflicted on him."

"But the patient will certainly die if something be not done." With all deference, my enterprising friend, you must not say that so positively, and even if it were undoubtedly true, I must be allowed to add, it is none of your business. If you have any mode or means of relief for the sufferer, proceed, at once, to employ it, under the general responsibility of the healing-art—responsibilities the force of which can neither be augmented nor diminished by your conjectures about the duration of the patient's life. The Supreme author and arbiter of life has reserved all knowledge, on that subject, to himself; but has given a solemn lesson of its sacredness in his sight, in that august command—addressed as well to the surgeon as to the highwayman—"Thou shalt not kill."

"No surgery elicits praise, but it consists of *exploits, achievements, feats*—not exactly that of leger-de-main, but, if I may coin a word for my purpose, of *sanglant-de-main*. And as to surgeons themselves, if one cannot have himself called a *bold*, or a *fearless*, or a *daring* practitioner, he had better have nothing at all said of him. All beside belongs to that faint praise that is proverbially damning. I have lately read a surgical essay of considerable pretensions, in which you can hardly find any other eulogistic adjectives than those heroic ones I have just repeated. A sober, unambitious phraseology seems to have been as repugnant to the author as it was to a poetic class-mate of mine, whose recitations were made amusing by the unvarying loftiness of his diction, never condescending to translate the word *equus*, for example, even if it occurred in a plowing lesson of a Bucolic, into any thing but a *steed* or a *courser* or a *charger*.

Indeed, however, surgery is not an heroism; but a calm and beneficent philosophy. Little does he seem to need boldness, or courage or daring, who has to deal only with the poor, trembling, feeble, despondent victims of disease. The duties of the surgeon often demand firmness, self-possession, composure, in the highest degree: but he must be a fiend, who, over the prostrate and confiding subject of his painful ministrations, can be actuated by those vulgar and heartless qualities for which your daring operator is wont to be extolled. The ordinary current of surgical duties, not bearing the captivating language of an epic, so well even as my friend's *equus* did, pursues its healing course, without observation, while its babbling tributary, addressing the senses and imagination, and enlisting the vocabulary of the passions, concentrates upon itself the popular regard.

'But boldness,' says Mr. John Bell, 'is a seducing word, and the passion of acquiring character, in operations, is surely full of danger: it is fit for those only to profess, who have no higher claim to the public esteem. We are but too apt to allow the '*audax in periculis*,' to be the character of a good surgeon. But this is a temper of mind, and a line of conduct, which can benefit nothing but the character of the surgeon himself: for as to his patient, this shameless thirst of fame, this unprincipled ambition, is full of danger."

"At the time when Hunter commenced his career in London, Hawkins had 'taken the town' as an operator. The one invented a modification of the gorget, and rested his fame upon its expert employment—the other investigated the laws of life and disease, and became the Solon of his profession. The former is forgotten, except to the curious inquisitor of the past, while the latter is as familiar to the rising generations of disciples all over the world, as he once was to the students of St. Geoge's, and venerated as he is familiar."

Our limited space will not permit us the gratification of further extracts. Some *faint* idea of the force and spirit of Dr. Flint's Report, may however be gained from the above.

---

*Prize Essay, read before the Ohio State Medical Society, at its Eighth Annual Meeting in Dayton, June* 1853. By SAMUEL G. ARMOR, M. D.

Dr. Armor discusses in his Essay the zymotic theory of essential fevers and other disordered conditions of the blood, taking as his leading proposition, Liebig's statement that "the chemical force and the vital principle hold each other in such perfect equilibrium that every disturbance, however trifling, or from whatever cause it may proceed, effects a change in the blood."

The subject is treated with ability, and shows considerable acquaintance with the chemico-physiological doctrines of the day.

---

*On the use and abuse of Alcoholic Liquors in Health and Disease.* By WM. B. CARPENTER, M. D., F. R. S. *With a Preface.* By D. F. CONDIE, M. D. Blanchard & Lea.

In his preface to this edition, Dr. Condie says, "Although we may differ from him (the author) as to the value of alcoholic drinks as a remedy for the cure of disease, still, in all his leading conclusions, as to the effects of these liquors upon the corporeal, mental and moral functions of the healthy human system, he is fully borne out by the results of our own observations and experience, acquired during a long series of years' practice as a physician, and as an active participator in the effort at temperance reform, which, originating with a few philanthropists in the United States, speedily enlisted the co-operation of 'thousands of the best and most talented individuals' of our own and other lands."

"To popularise, as far as possible this edition, the various technical phrases which occur in it have been explained, so as to render their meaning familiar to the unscientific reader."

With these explanations, this Essay will become the most popular, as it already is the most scientific text-book of temperance now extant.

